# A Scoping Review on Movement, Neurobiology and Functional Deficits in Dyslexia: Suggestions for a Three-Fold Integrated Perspective

**DOI:** 10.3390/ijerph20043315

**Published:** 2023-02-14

**Authors:** Michele Pellegrino, Tal Dotan Ben-Soussan, Patrizio Paoletti

**Affiliations:** Research Institute for Neuroscience, Education and Didactics, Patrizio Paoletti Foundation for Development and Communication, 06081 Assisi, Italy

**Keywords:** dyslexia, cerebellum, quadrato motor training, neuroplasticity, neurorehabilitation

## Abstract

Developmental dyslexia is a common complex neurodevelopmental disorder. Many theories and models tried to explain its symptomatology and find ways to improve poor reading abilities. The aim of this scoping review is to summarize current findings and several approaches and theories, focusing on the interconnectedness between motion, emotion and cognition and their connection to dyslexia. Consequently, we present first a brief overview of the main theories and models regarding dyslexia and its proposed neural correlates, with a particular focus on cerebellar regions and their involvement in this disorder. After examining different types of intervention programs and remedial training, we highlight the effects of a specific structured sensorimotor intervention named Quadrato Motor Training (QMT). QMT utilizes several cognitive and motor functions known to be relevant in developmental dyslexia. We introduce its potential beneficial effects on reading skills, including working memory, coordination and attention. We sum its effects ranging from behavioral to functional, structural and neuroplastic, especially in relation to dyslexia. We report several recent studies that employed this training technique with dyslexic participants, discussing the specific features that distinguish it from other training within the specific framework of the Sphere Model of Consciousness. Finally, we advocate for a new perspective on developmental dyslexia integrating motion, emotion and cognition to fully encompass this complex disorder.

## 1. Introduction

*“Reading is not exhausted merely by decoding the written word or written language, but rather anticipated by and extending into knowledge of the world. Language and reality are dynamically intertwined”*

[1] (p. 5).

This quote by renowned pedagogist Paulo Freire highlights how reading is essential for discovering the world and opening up to it. While reading was once an advantage of the privileged few, it has become a basic skill necessary for everyday life. Progress in mastering reading and writing skills during the early school years is often taken for granted. However, for people with dyslexia, these skills are not acquired in the usual manner, and the subsequent difficulties in keeping up with others can have challenging consequences for their well-being, quality of life, and academic achievement. Furthermore, longitudinal studies indicate that dyslexia is a chronic condition that persists well into adulthood [2] and, thus, could have lifelong detrimental effects. Although one of the first described cases of individuals with dyslexia can be traced back to 1676 [3], a more targeted examination of reading disabilities in children was described for the first time in seminal studies in 1896 and 1897, respectively, by Pringle-Morgan [4] and Kerr [5]. The difficulties were labeled “dyslexia” because of the similar reading deficits found following brain injury among previously skilled adult readers (dys-: difficult; lexis: speech). Currently, the worldwide estimated prevalence of developmental dyslexia (hereafter referred to as dyslexia) falls in the range from 3% to 7% [6,7,8].

Dyslexia has been defined as “*deficient literacy acquisition despite adequate intellectual ability and sufficient educational exposure*” [9]. Another widely used definition comes from a consensus between the International Dyslexia Association (IDA), the National Center for Learning Disabilities (NCLD) and the National Institute for Child Health and Human Development (NICHD), which define dyslexia as [10] (p. 2): “*…a specific learning disability that is neurobiological in origin. It is characterized by difficulties with accurate and/or fluent word recognition and by poor spelling and decoding abilities. These difficulties typically result from a deficit in the phonological component of language that is often unexpected in relation to other cognitive abilities and the provision of effective classroom instruction. Secondary consequences may include problems in reading comprehension and reduced reading experience that can impede growth of vocabulary and background knowledge*”. Accordingly, extensive research in recent years has established dyslexia as not only a specific learning disorder but also a neurobiological syndrome [11]. Indeed, while reading deficits are the primary diagnostic criterion, dyslexic individuals can display a variety of other difficulties, such as the impaired ability to sequence word sounds auditorily and letters visually, poor short-term memory for sequences, and poor motor sequencing leading to incoordination, clumsiness and poor handwriting [12]. Since sensorimotor deficits are often also observed in different developmental disorders, some researchers attribute dyslexia’s cognitive and motor deficiencies to both functional and structural abnormalities of the cerebellum [13,14].

**Figure 1:**
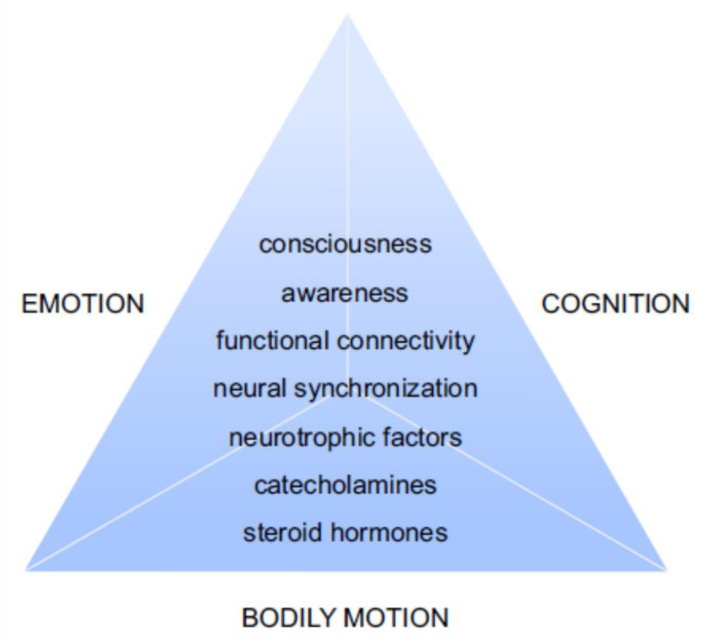
The interconnectedness between motion, emotion and cognition and the possible dimensions that need to be examined in order to achieve a comprehensive overview of the effects of training. Adapted from [17,18].

The next sections will explore existing models and treatments for dyslexia; we will start addressing different models and theories about the etiology of the disorder, with a particular focus on the cerebellar theory and on symptoms such as poor coordination and praxic abilities (Section 2). We will then focus on the cerebral and especially cerebellar anatomical differences between the normal reading and dyslexic participant and their involvement in affective processes (Section 3). In particular, we will examine the cerebellar alpha oscillatory activity as the connecting point between structural and functional changes. We will highlight the importance of an integrated sensorimotor, emotional and cognitive point of view in approaching this complex neurobiological disorder (Figure 1) while examining different types of interventions (Section 4). We will explore the possibility of addressing these three aspects through a specific form of movement meditation, the Quadrato Motor Training (QMT) (see [15,16] for reviews) reporting recent studies utilizing QMT among those with dyslexia (Section 5). Finally, we will discuss the presented results of QMT within the framework of the Sphere Model of Consciousness, a neurodevelopmental model of consciousness and an interesting key to interpretation for QMT’s results in dyslexic participants.

Let us start with the leading models and interpretations of the disorder.

## 2. What Is Dyslexia: Models and Neuronal Correlates

### 2.1. Models and Interpretations of the Disorder

Dyslexia is a complex neurodevelopmental disorder, and as such, it has attracted the interest of many researchers, many of whom theorized about its etiology and attempted to characterize its symptoms. Studies of dyslexia generally report a wide array of deficits in vision, attention, auditory and temporal processes, phonology, and language [19,20,21,22,23]. More specifically, dyslexic individuals exhibit difficulties with word recognition and spelling despite adequate instruction, intelligence, and intact sensory abilities [10]. Dyslexia is also characterized by difficulties in decoding during reading, whereas by comparison, comprehension during listening is typically more intact [7].

Over time, different models have been proposed to explain these symptoms, trying to trace them back to a common underlying deficit (or deficits). Two of the most commonly referenced models are the phonological deficit theory [24,25,26,27] and the magnocellular deficit theory [19,28,29]. The phonological deficit theory posits that dyslexia’s reading difficulties derive from problems in breaking down spoken words into their constituent sounds (syllables or phonemes), while the magnocellular deficit theory proposes that its symptoms originate from abnormalities in basic sensory and motor processing and that phonological impairments are secondary [19,30]. Although these sensorimotor abnormalities are thought to be related to visual and auditory magnocellular systems, they also extend to vestibular and motor systems [19]. It is well known that people affected by dyslexia tend to be clumsy and uncoordinated and have poor handwriting, balance, and many other “soft” cerebellar signs, such as “reach and gaze overshoot” and muscle hypotonia [31]. In addition, dyslexic children display impairment in continuous tapping tasks compared to normal readers [32,33]. This is thought to reflect a deficit in timing precision on bimanual tasks that require the integration of asynchronous responses. As timing precision is a function attributed to the cerebellum [34,35], there is evidence that reading problems can be traced back to a fundamental sensorimotor cause.

Such a critical sensorimotor deficit in dyslexia symptomatology is the basis of the cerebellar deficit theory [13], as the cerebellum plays a major role in the processing speed and the temporal coding of multisensory inputs [36]. Indeed, Nicolson et al. [13] observed that impaired phonological processing could not sufficiently explain the wide array of sensorimotor and neuromotor impairments seen in dyslexia, ranging from motor coordination and balance to information processing speed and implicit motor learning [13,30,37,38,39,40,41,42,43,44,45,46,47,48,49,50]. Consequently, they proposed that cerebellar dysfunction and impaired procedural learning may explain both symptoms primarily related to reading as well as the broader range of deficits observed in dyslexia [13,51]. Accordingly, the theory linked “*cerebellar dysfunction to the phonological difficulties in dyslexia via the articulatory system; visual sequencing problems to the cerebellar role in visual attention and eye movements; incoordination, clumsiness, and poor handwriting to the cerebellar contribution to motor control, and the role of the cerebellum in implicit learning to the slow, laborious learning seen in dyslexic individuals*” [12] (p. 270). Evidence supporting this conceptualization (of functional interactions between motor control systems, language and reading) can be found not only in neuropsychological test findings that highlight poor cerebellar processing among many children affected by dyslexia [51,52] (for reviews, see [53,54]), but also from neurophysiological studies [55,56] (for reviews see [57,58]). Eckert and colleagues [59], for example, reported decreased right cerebellar volume among dyslexics compared to controls; this abnormality was correlated with difficulties in reading, spelling, and language processing. Thus, the involvement of the cerebellum in language seems to be well-established.

Accordingly, the etiology of dyslexia appears complex and multifactorial, as reflected by recent neuropsychological and neurobiological accounts of the disorder that are also increasingly comprehensive and attentive to various underlying factors [7]. In particular, recent research has tried to unearth fundamental dysfunction in visual, auditory and motor domains [7,30] as well as establish what type of model (single-deficit or multiple-deficit) may better explain dyslexia symptoms using different kinds of computational and non-computational modeling [60,61,62].

Further, the deficits that appear when dyslexic individuals attempt to execute simple and complex neuromotor acts, combined with the presence of clinical signs (such as dysrhythmia and synkinetic movements) [63,64,65,66,67], have historically all been considered related to the abnormal temporal organization of motor skills [67,68]. However, considering the additional impairments found in executive functions such as programming movements, in visual and kinaesthetic sensory processes, and in the capacity to evaluate their own performance and correct their errors, Chiarenza [69] advanced the hypothesis that dyslexia is not only a phonological deficit but “*is a more complex disorder associated with a malfunction of higher cognitive functions, including attention, phonological analysis, verbal-motor coordination, control mechanisms and feedback, and memory*” [70]. Further, he asserted that these malfunctions seem to be closely and causally related to each other and that the main impairment in dyslexia involves praxic abilities [70]. In support of this hypothesis, Chiarenza demonstrated that dyslexic children showed a latency delay and reduced amplitude of movement-related potentials in various cerebral areas during the same skilled motor task [71,72] (for a very recent review on quantitative EEG in dyslexia, see also [73]). Therefore, the author hypothesized that dyslexia symptoms could be caused by a timing defect that causes an integration deficit and subsequent dysfunction among numerous hierarchically organized processes occurring at different levels and times [70].

In a similar way, Lachmann and colleagues also argued that dyslexia might entail more complex alterations in the processing of the stimuli. More specifically, reading deficits may be a result of a failed integration of information with the sufficient speed and accuracy required for reading. In other words, dyslexic children may fail to adequately differentiate the visual processing of linguistic and nonlinguistic stimuli. This could lead to improved performance in certain visual tasks and normal performance in others, but lower performance in reading tasks compared to controls [74,75].

While all models and interpretations of dyslexia introduced thus far have their merits and explanatory power, a universally shared consensus on dyslexia’s etiology has yet to be reached.

Furthermore, one area that is often left aside in favor of examining more cognitive symptoms is how dyslexia can affect emotional and affective processes in children, adolescents and adults. Detrimental emotional effects of dyslexia are often considered only as secondary and caused by poor academic performances [76], while the emotional components of executive functions eventually affected are neglected. Executive functions are, indeed, traditionally considered purely cognitive processes, but an alternative distinction has been proposed in relatively recent years between “cool executive functions”, abstract and decontextualized tasks lacking emotional or motivational components [77,78], and “hot executive functions” [79] with clear affective and motivational components, such as beliefs, desires, reward and punishment, social behavior, and the emotional components of decision-making [80]. In order to have a clear picture of problems encountered by people affected by dyslexia, this fundamental distinction seems to be crucial.

Emotional malfunctions may be at the roots of affective problems, such as frustration, low self-esteem, and even anxiety and behavioral problems, which can become serious primary clinical problems in combination with well-known reading difficulties [81,82,83,84,85,86,87,88]. For this reason, it is very important trying to combine the existing models focusing on cognitive and bodily processes involved in dyslexia with these emotional aspects, which are crucial for the well-being of people affected by this disorder.

### 2.2. Neural Correlates of Dyslexia

Given the complex neuropsychological profile of this disorder, many studies have investigated dyslexia using a different approach, namely, searching for structural and functional differences in the dyslexic brain compared to normal-reading individuals. As the most characterizing deficit of dyslexia, the phonological deficit has been found to be associated with abnormalities in cortical response and asymmetrical activity in frontal and temporal language/reading areas [27,89,90]. In particular, reduced activity has been identified in a left temporoparietal region (related to phonological processing and phoneme-grapheme conversion) and in a left occipitotemporal area (linked to whole-word recognition) (for a review, see [7]). Moreover, during reading and phonologic processing tasks, reduced blood flow in the left temporoparietal area has been found among adult dyslexics compared to normal readers in PET studies [91,92,93], while normal activation was found in left inferior frontal areas [91,92]. In addition, the enhanced asymmetrical activity in the left hemisphere observed in normal readers, evidenced by fMRI studies, for instance, in which normal readers demonstrated increasingly higher activation in temporoparietal areas in relation to increasing demands for phonological processing [94,95] is in contrast to the experience of adult dyslexic readers, in whom this activation has been found significantly attenuated during reading tasks [96].

These fronto-temporal and cerebellar functional abnormalities identified in individuals with dyslexia are further corroborated by results coming from structural imaging studies. Raschle and colleagues [97] reported decreased grey matter (GM) in the frontal and temporal networks of dyslexics, while local white matter (WM) change was demonstrated in left temporoparietal regions and in the left inferior frontal gyrus [98,99,100], areas corresponding to phonological skills (for a review, see [7]). Structural imaging also demonstrated lower cerebellar declive volume (associated with impaired reading abilities; [101]) and, as mentioned before, Eckert and colleagues [59] found the volume of the right anterior lobe of the cerebellum significantly correlated with reading performances.

The extent of the differences between the dyslexic brain and the normal reading brain is not limited to these structures. Dyslexic individuals can differ from controls across a wide array of brain areas, including the insula, caudate, corpus callosum, left temporal lobe, and thalamus [102,103,104,105,106,107,108]. As an example, the insula is thought to be involved in the auditory temporal processing of non-linguistic auditory stimuli, and the aforementioned area constitutes a neural correlate of deficit temporal processing of speech and nonspeech sounds in dyslexia [109]. Nonetheless, the cerebellar and frontal differences between dyslexics and controls are by far the most consistently reported (for reviews, see [101,110]). As Eckert concluded in his 2004 review [111], the main regions that display structural changes in dyslexia are the inferior parietal lobule, the inferior frontal gyrus and the cerebellum.

Given its relevance in dyslexia, in the next section, we will briefly examine cerebellar deficits and impairments characterizing dyslexia.

## 3. Reading the Cerebellum

### 3.1. The Cerebellum: From an Exclusively Motor Area to a Cognitive One

The incredible and complex organization that is the cerebellum is thought to be the basis of many different functions, the most known and studied of which is the generation of internal models of the sensorimotor system [112,113], which can be then used to predict [114], adjust, and optimize the outcomes of motor commands [12]. Historically, the cerebellum was thought to be exclusively or primarily responsible for the planning and execution of movements. Yet, following the seminal work of Leiner and colleagues [115], and after some initial resistance from the scientific community (for reviews, see [54,116]), the cerebellum was finally reframed as an important structure for human cognition and emotion. Studies found that the cerebellar response could be attenuated by automating a task but keeping motor demands constant [117] or be modified by reducing cognitive demands [118].

Further proof in support of the cognitive role of the cerebellum came from the study by Kim and colleagues [119], which demonstrated through functional MRI that the dentate region of the cerebellum showed increased activation during cognitive processing. This is particularly interesting because the caudate represents the major output structure of the dorsolateral prefrontal cortex [120,121] (for a review on cerebellar projections and non-motor functions, see also [122]), an area known to be involved in general executive control functions, and in particular “cool” executive functions [123,124,125] (for a recent review, see also [126]). Finally, in 2008, Ito theorized that the role of the cerebellum in cognitive functions mirrored its role in motor functions. According to this theory, the skillful execution of the mental process is possible thanks to feedback mechanisms and internal representation allowed by cerebellar activity [127]. Moreover, there is consistent evidence that several co-activations occur between cerebellar structures and prefrontal cortices while performing different cognitive functions. In particular, increased activation in both the dorsolateral prefrontal cortex and cerebellum has been found while administering the Wisconsin card sorting test, a typical test for “cool” executive functions [128,129] and with many working memory tasks [130,131,132,133,134,135,136,137]. As concluded in a review by Diamond [138], these two areas are strongly linked: “*The cerebellum and prefrontal cortex participate as critical parts of a neural circuit that is important when (1) a cognitive task is difficult as opposed to easy, (2) a cognitive task is new as opposed to familiar and practiced, (3) conditions of the cognitive task change, as opposed to when they remain stable and predictable, (4) a quick response is required, as opposed to longer response latencies being acceptable, and (5) one must concentrate instead of being able to operate on automatic pilot*” [138] (p. 45).

A relevant question to be asked is: How does the cerebellum communicate with prefrontal areas within this network?

It has been proposed that the cerebellar alpha oscillatory activity could be the underlying neural mechanism of cerebro-cerebellar crosstalk, particularly regarding planning and movement execution, reading, and language processing [139,140,141]. Cerebellar alpha oscillations may mediate both cortical and cerebellar communication [140,142]. This is supported by previous studies showing sensorimotor cerebellar stimulation may actually modulate firing patterns in frontal regions that are crucial for cognitive functions, such as planning and creativity [143,144,145].

It should be noted that cerebellar lesions have effects beyond motor functions and cognitive processes. In 1998, Schmahmann and Sherman noted a consistent pattern of deficits and impairments characterizing patients with cerebellar lesions: disturbances in both “cool” executive functions (e.g., deficient planning, set-shifting, abstract reasoning, working memory, and decreased verbal fluency); impaired spatial cognition (e.g., visuo-spatial disorganization and visual-spatial memory), “hot” executive functions, such as emotion-related changes (e.g., flattening or blunting of effect, disinhibited or inappropriate behavior), in addition to language problems (e.g., dysprosodia, agrammatism and mild anomia) [146]. Schmahmann and Sherman [146] termed this clinical pattern the Cerebellar Cognitive Affective Syndrome (CCAS) in light of the widespread effects of these lesions on motor, cognitive and affective processes.

In summary, consistent with the proposed role of the cerebellum in cognition, this structure forms close connections with frontal and prefrontal brain areas, providing the neural basis through which it contributes to neocortical information processing (for a review, see [145]). As we briefly covered earlier, both the cerebellum and the prefrontal cortex play a crucial role in the neural network, especially during cognitively demanding and novel tasks [138]. These findings lead us to consider the role of the cerebellum as not limited to regulating the rate, force, rhythm, and accuracy of movements but also the speed, capacity, consistency, and appropriateness of both cognitive [54,147,148] and affective processes [146]. Further, it highlights the importance of an integrated examination of subcortical regions during cognitive tasks [138,149,150,151] and cerebellar oscillatory activity in communicating with neocortical areas [140,142]. For all these reasons, the current consensus on the role of the cerebellum is that the triad consisting of motor, cognitive and affective functions is fundamentally intertwined in this complex and fascinating structure [138,146].

### 3.2. Cerebellar Involvement in Reading and Dyslexia

Functional imaging studies have shown activation in the left inferior frontal and right cerebellar hemispheres during fluency tasks, verb generation tasks, passive listening to clicks, linguistic working memory tasks and rapid production of verbal stimuli [117,135,152,153,154]. In addition to this evidence suggesting a functional role of the cerebellum in language processing, a wealth of studies exist supporting this link [155,156,157,158]. Moreover, a meta-analysis of the functional topography of the cerebellum identified activation in right cerebellar regions, including lobules VI and crus I/II, during language processing, thus further reinforcing the cerebellar role in language [57]. Booth and colleagues [159] also provided evidence for reciprocal functional connections between the cerebellum and the left inferior frontal and left lateral temporal regions, two areas already known to be involved in language processing. While the involvement of the cerebellum during language processing seems clear, what is its precise involvement during reading?

Reading-related activity in the cerebellum seems to be focused on lobules VI and VII, reaching their maximum in the right posterolateral cerebellum [160,161]. It is evident that these are the same cerebellar areas activated during language tasks [57]. The precise localizations of activation depend on the demands of the particular reading task, but the most consistent activation has been found in the left, the right, or bilateral lobules V, VI and VII (for a review, see [12]). Cerebellar involvement in reading is also highlighted by differences between impaired and normal readers. Normal readers usually show right-lateralized cerebellar asymmetry, while dyslexic individuals usually show symmetry that is correlated with phonological processing deficits [106,162]. Although reduced cerebellar size and asymmetry may be related to general cognitive deficits and not only to dyslexia [163], many studies demonstrated cerebellar abnormalities among dyslexic individuals. The results from Eckert and colleagues [59] echo that of Leonard and colleagues [105], who found the right anterior cerebellum smaller in dyslexic adults. Structural imaging further confirms, although not consistently [164,165], that the dyslexic brain shows significantly reduced grey matter in cerebellar nuclei, lateral lobule VII, and the anterior cerebellum [104,166,167]. Similar to the structural findings, the anterior lobe of the cerebellum has also evidenced reduced activation in dyslexics [168,169], as well as alterations in functional connectivity between this area and the angular and inferior frontal gyri [170,171], two brain regions critical to reading and phonological processes.

A possible explanation for the cerebellar involvement in dyslexia could lie in the cerebellum’s role as a source of alpha activity within the cerebro-cerebellar network, a neural network critical for reading. This could align with the proposed role of an impaired timing process in dyslexia. We will explore in the next sections how these anomalies could be reduced and “normalized” using different types of treatment. We will particularly focus in detail on the benefits, or lack thereof, to be obtained from physical training.

## 4. Interventions, Neuroplasticity and Dyslexia

In the next sections, we examine studies investigating different types of interventions in children affected by dyslexia, the effects of sensorimotor training in dyslexic participants and linked functional and structural changes, and all the available literature existing on the QMT. Consulted databases were PsycInfo, PubMed and Google Scholar. This resulted in a total of 27 non-theoretical, non-review/meta-analysis studies being included. A cumulative sample of 241 dyslexic participants and 195 children with a familial risk of dyslexia were included. In addition, counted separately, all the examined studies on the QMT yielded a total of 608 participants (23 with dyslexia and 585 healthy participants).

Dyslexia has long been considered a chronic reading disorder in which a number of symptoms from childhood can persist into adulthood [172,173,174,175], regardless of language transparency [176,177]. Over the years, different types of interventions have been advanced, trying to find ways to reduce or eliminate symptoms of dyslexia. Traditional interventions, such as direct teaching, have garnered a solid base of evidence for their efficacy, but many alternative therapies for dyslexia exist, with different degrees of success [178]. The most effective interventions seem to be those that provide intensive and explicit instruction in phoneme awareness, reading fluency, and reading comprehension [179], thus primarily addressing the typical phonological deficits of dyslexia. Torgesen and colleagues [180,181] demonstrated that phonological problems could be remediated with relatively short periods of intensive remedial instruction. For example, the authors contrasted two different treatment approaches among dyslexic children: (1) a multisensory, bottom–up, explicit approach for developing phonemic awareness, and phonemic decoding/encoding skills with minimal text instruction and (2) and embedded phonics instruction approach. The outcome of this intensive experiment was a general improvement in reading and phonemic decoding, regardless of the type of intervention. However, while almost all children displayed better results after the intervention, only half of the outcomes remained stable or continued to improve during follow-up, and some children even lost the acquired benefits [181]. In a similar way, a study by Simos and colleagues [182] showed that all children in their cohort displayed a typical dyslexia-specific profile of weak activity in the left posterior superior temporal gyrus and inferior parietal areas, and strong activation of homologous regions in the right hemisphere in the pre-intervention phase. The authors demonstrated how completion of a phonologically based reading program resulted in consistent improvement in phonologic decoding abilities and normalization of the brain activation profiles for all of the children [182].

While such intervention programs have been phonologically focused, there is a wealth of data relating to other types of deficits. Thus, in creating, evaluating, and administering treatments, factors beyond language intervention should be considered to achieve optimal outcomes. For example, “cool” executive functions, such as attention and working memory, should be assessed and treated [183], together with “hot” executive functions and emotional aspects, such as low self-esteem, anxiety and behavioural regulation, to fully encompass the well-being of people with dyslexia [81,82,83,84,85,86,87,88]. A recent study [184] implemented a remedial technique for dyslexia the consisted of asking children to repeatedly read texts aloud while listening to a song (reading with vocal musical masking, or RVM; [185]). According to Leloup and colleagues [184], their hypothesis was that this intervention would improve executive skills rather than directly reinforcing specific coding skills, leading to improved reading abilities. Their findings indeed supported their hypothesis, that processing speed and phonemic fluency were the best predictors of improved reading skills related to the remedial intervention [184]. In a related vein, other studies examined interventions that tapped into a wider array of functions, demonstrating the role of music-making and listening as a potential tool in the remediation of dyslexia symptoms [186,187,188,189] and also having some deep effect on improving mood and elicit positive emotions (for a review on brain correlates and possible applications of music in treating neurological and psychiatric disorder see [190]). In particular, the act of practicing an instrument, which requires multi-sensory, motor integration, emotional engagement and creativity, may compensate for dyslexic deficits, such as poor working memory and accuracy during reading [187,188] eliciting changes in the central and peripheral nervous systems that correlate with improvements in motor, auditory, and learning abilities [191,192]. Such improvements related to musical training (i.e., piano lessons) were described in a case study by Eren [193] demonstrating that, after 8 months of instrument training, the dyslexic participant showed not only strengthened attention and memory, but also higher confidence, better behavioral regulation, and generally more positive emotions [193].

More generally, the link between physical activity, motor skills and dyslexia has been investigated by different authors in the last 20 years. While motor ability can be further clarified as fine and gross motor skills [194,195], which may, in turn, influence language and reading skills in different ways, here we will mostly focus on gross motor skills. For example, in 2001, Lyytinen and colleagues compared children with and without familial risk for dyslexia from birth to five years old on a series of variables pertaining to motor skills and language development. They attempted to identify potential predictor variables for dyslexia and found that early vocalization and motor development, especially gross motor skills, were linked to later language development [196]. Along similar lines, another longitudinal study highlighted how children at risk for dyslexia and with slower motor development showed a smaller vocabulary and produced shorter sentences compared to other children [197]. In addition, from also a cross-sectional perspective, dyslexic children were found to have lower performance in balance tasks compared to controls [198].

Furthermore, physical activity has been found to be linked to cognitive functioning in children with learning disabilities [199]. This meta-analysis found a significant positive relationship between physical activity and cognitive functioning, suggesting that physical activity can be beneficial both for children with learning disabilities and with healthy children, highlighting the importance of physical activity for their well-being [199].

One reason for positive outcomes using sensorimotor interventions in the remediation of dyslexic deficits and the connection between language acquisition and motor skills could lie in the aforementioned role of cerebellar circuits in phonological processing via articulatory monitoring [31,200]. For this reason, Stoodley and Stein [12] (p. 271) suggested that “*cerebellar circuits may be particularly important for compensation and remediation of reading difficulties*”. Consequently, studies aimed to investigate possible improvements in reading through integrated sensory stimulation and visuo-motor and vestibular exercises [201,202]. Although these results are compelling, the effectiveness of such evidence-based intervention programs is often debated [90,201,202,203,204,205].

**Figure 2:**
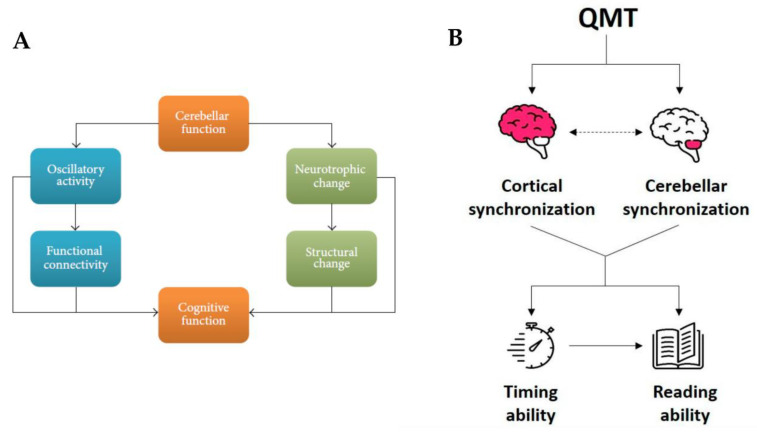
(**A**) Interconnected relationship between cerebellar and cognitive function. The relationship is mediated via two interrelated routes. The first is slow rhythm oscillations, manifested in functional connectivity; the second is molecular effects on structural changes in connectivity. Adapted from [15]. (**B**) Effects of the QMT on both timing and reading abilities mediated by the interconnected relationship between cortical and cerebellar synchronization. Adapted from [16].

A suggestion on how to explore multiple levels of the effects of sensorimotor training comes from a recent model presented by Ben-Soussan and colleagues [15] which outlined the importance of the cerebellar activity in higher cognitive functions and examined its influence on different levels of brain organization (Figure 2). The authors suggested the presence of two intertwined pathways leading to functional and structural changes, one related to alterations in cerebellar oscillatory activity and the other, with a longer time frame, related to neurotrophic factors and neuroplasticity [15]. Both pathways, and their main preliminary starting point (i.e., the cerebellar activity), are altered in dyslexia and other neurodevelopmental disorders [206,207,208].

Its focus on cerebellar involvement, neurobiological factors and the junction between functional and structural pathways makes Ben-Soussan and colleagues’ model [15] a novel alternative way to integrate both magnocellular and cerebellar theories of dyslexia. Moreover, the processes it emphasizes, which connect cerebellar to cognitive functions, could help in interpreting and understanding the complex dynamics of dyslexia’s deficits. The model is mainly based on two lines of evidence. The first relates to the functional level and the many already mentioned reports suggesting cerebellar alpha oscillatory activity as the neural system underlying the planning and execution of movements as well as reading and language comprehension [139,140,141].

The second line of evidence pertains to an anatomical and structural level, particularly focusing on Brain-Derived Neurotrophic Factor (BDNF) and Nerve Growth Factor (NGF) from the neurotrophin family. Neurotrophins, and their precursors, pro-neurotrophins, can influence mature and developing neural circuits in different ways [209,210]. For example, ProBDNF and NGF have been linked to learning, spatial cognition and neuronal plasticity [211,212,213,214], while proNGF is related to nociceptors, neuronal death and neurodegeneration [215]. Importantly, BDNF has been found to be a crucial factor in mediating neuronal connectivity and movement-dependent plasticity in the cerebellum [216,217]. Further, among human participants, functional connectivity changes induced by sensorimotor training were found to be BDNF-dependent [218,219]. In the same way, animal models also demonstrated a relationship between BDNF and cerebellar functioning, identifying important cerebellar deficits in BDNF knockout mice [220,221]. Moreover, numerous animal models have demonstrated training-induced effects in the motor cortex, the basal ganglia and the cerebellum (for reviews, see [218,219,222,223]).

Nevertheless, human studies examining motor training-induced changes in cognitive functions have generally neglected to study motor-related brain areas. Such studies have almost exclusively concentrated on frontal and prefrontal areas, reporting an increase in frontal alpha synchronization after motor training [143,144,224]. The model by Ben-Soussan and co-workers [15] (p. 3) suggests that “*motor training in humans may serve as an optimal model to test the possible cerebellar cognition relationship in two interrelated routes: (1) sensorimotor training may result in fast occurring slow frequency oscillatory modulation leading to improved cognitive performance. (2) Cerebellar changes can be further stimulated through training by activating molecular mechanisms (e.g., neurotrophins*)”. Interestingly, the importance of cerebellar oscillatory function in neuroplasticity [225,226] has long been acknowledged in studies related to motor learning. A couple of studies [227,228] have already demonstrated cerebellar microstructural changes following sensorimotor training. Thus, techniques such as sensorimotor training paradigms may efficiently achieve beneficial effects in the treatment of dyslexia impairments such as the decreased right cerebellar volume reported by Eckert and colleagues among dyslexics compared to controls [59] (see Section 2.1), through the dual functional and structural pathways highlighted in this model [15]. As we said before, considering neuropsychological and neurobiological components of this developmental disorder is important to understand its causes and symptoms, but we must not fail to consider something central for dyslexic people: their emotional well-being.

Indeed, in the next section, we will examine outcomes of the Quadrato Motor Training (QMT), which obtained promising results in the remediation of dyslexia symptoms and in improving cognitive and emotional aspects, thus intervening on the full spectrum of motion, emotion and cognition.

## 5. Quadrato Motor Training and Dyslexia

### 5.1. What Is Quadrato Motor Training?

**Figure 3:**
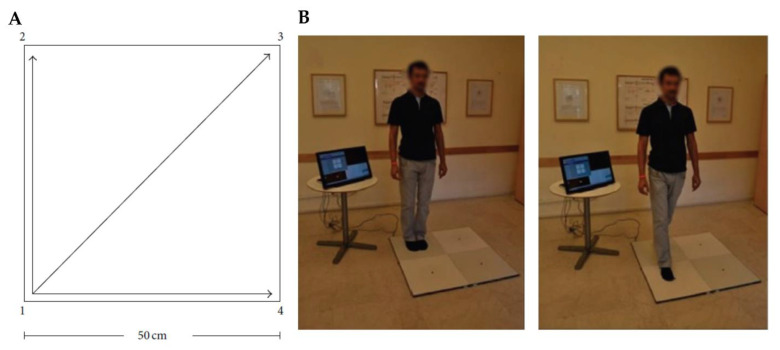
Quadrato Motor Training (QMT). (**A**) A graphical illustration of QMT. (**B**) A participant waiting for the next QMT instruction (left) and following the QMT instruction (right).

Quadrato Motor Training (QMT) is a non-aerobic, coordination-demanding, and cognitively engaging movement practice [229,230]. Participants are required to alternate between movements and static postures while focusing attention and awareness on their bodies in the present moment and excluding possible distractions. QMT is conducted on a 50 × 50 cm square known as the Quadrato space. Its corners are labeled with the numbers 1–4. Participants are required to either produce or inhibit a motor response in the Quadrato space on the basis of specific verbal instructions presented. The motor responses are steps in one of three possible directions: right or left; forward or backward; or diagonally (e.g., a verbal instruction can be “1–2,” which directs the practitioner to take a step forward from corner number 1 to corner number 2). When two numbers of the verbal instruction are the same (e.g., “2–2”), participants have to inhibit the impulse to move upon hearing the voice command and wait for the next instruction. The inhibitory control (cognitive and motor) required to make a decision based on cognitively processed information related to the specific verbal instruction is one of the main features of the QMT. Inhibitory control is also involved in the instruction to keep their eyes focused straight ahead with their hands by their sides and to follow the next instruction without stopping even if they make any errors (Figure 3) [16,230].

QMT requires executive functions such as motor coordination, balance, awareness of the body and its location in space, alongside cognitive elaboration and error monitoring, as well as regulation of behavior involving emotional content [230]. Thus, it involves executive functions related to cognitive, emotional and motor dimensions and, in particular, working memory processes. It is important to note that working memory, especially verbal memory, is known to be lacking among dyslexic individuals [231,232,233,234]. Further, linguistic working memory tasks invoke cerebellar functions [135]. Therefore, at first glance, QMT seems to address some of the main functions compromised in dyslexia.

QMT can be defined as a mindful movement (MM) practice due to its capacity to bring focused awareness and concentration to the intricate experience of movement and the present moment [16,235,236,237]. MM practices require the involvement of important features of mindfulness, such as regulation of attention, working memory and preparation for execution of movements [236,238]. MM has been found to engage inhibition and response selection, as well as cognitive control and emotion regulation, requiring online sensorimotor updating [239,240]. The QMT fits very well within this description as it involves regulation of divided attention, execution of voluntary movement and regulation of behavior involving emotional content, alongside working memory updating and a need to be in the “here and now” due to constantly updating instruction commands [16,235]. Moreover, Diamond and Ling [241] underlined how MM can improve executive functions and promote neuroplasticity, directly affecting the praxic abilities identified by Chiarenza as peculiar to dyslexia [67] (see Section 2.1). We will now examine the current state of the literature about this particular form of MM.

### 5.2. Effects of Quadrato Motor Training

In the last ten years, many studies investigated different effects of QMT on varied samples of participants. These studies demonstrated how QMT can improve the cognitive and psycho-emotional functioning of its practitioners [17,229,235,237,242,243,244]. In particular, from a cognitive perspective, QMT induced gains in ideational flexibility which likely resulted from motor and cognitive inhibition. In fact, interventions with control groups involving only the cognitive component of QMT practice (only QMT’s verbal instructions) did not significantly change ideational flexibility [227,228]. Likewise, practicing a gross motor component similar to the QMT (i.e., taking steps) but without cognitive effort (i.e., reduced reaction choices) also did not change ideational flexibility [227,228,245]. Physical activity research indicates that more effective improvement in executive functioning resulted from combined cognitive, physical, and emotional engagement than on cognitive stimulation or physical activity alone [245,246], demonstrating the importance of jointly addressing both “hot” and “cool” executive functions. Moreover, QMT also enhances self-efficacy [93,229] and affects balance [229], which are both closely related to higher-order cognitive functions and self-regulation [247,248]. These results support the notion that cognitively engaging movement-based experiences can improve hot, as well as cool, EFs.

From an electro-physiological perspective, studies on healthy participants showed that QMT increases EEG power and coherence [229,249,250,251], mainly in the theta (4–8 Hz) and alpha (8–12 Hz) frequency bands. This is particularly interesting because increased intra- and inter-hemispheric functional connectivity in theta and alpha frequency bands is linked to improved cognitive functions and higher states of consciousness due to better integration of information and communication among different brain areas [252,253,254]. In addition, an increase in alpha power is thought to be related to an internally directed focus of attention [255,256,257,258] and has been found to be linked to increased creativity [144] and the experience of “flow” [259]. Thus, QMT seems to be strongly linked to internally oriented attention and, unsurprisingly, to increased reflectivity, mindfulness, and altered states of consciousness [235,260].

These electrophysiological and functional changes seem to be mirrored in neuroanatomical changes. Studies that focused on structural changes after QMT consistently found increased grey matter in the cerebellum and frontal lobe [15,242]. We have already highlighted how these two brain structures are crucial with regard to dyslexia and cognitive functioning in general, and how neuroimaging studies have found that their activation is closely coupled with each other [138,145,261]. Another important source of structural information comes from the study of white matter (WM). As mentioned above (see Section 2.2) changes in local WM were reported in left temporoparietal regions and in the left inferior frontal gyrus [98,99,100], areas corresponding to phonological skills (for a review, see [7]). Moreover, increased WM integrity is thought to be linked to improved functional connectivity and motor, cognitive and metacognitive performance [262]. Of particular interest for this work, many studies have shown that physical or meditative training can increase WM integrity [148,262,263,264,265,266,267,268,269,270,271]. Conversely, studies have shown that decreased WM integrity is associated not only with the aging process but also with psychiatric and neurological diseases [272,273]. It is important to note in this regard that QMT has already been shown to increase WM integrity in different cerebral and cerebellar regions [237,242,274]. Specifically, the QMT elicited increases in WM integrity in the cerebellar peduncles and the body of the corpus callosum, structures associated with cerebro-cerebellar and cerebellar communication, respectively [145,275]; QMT also elicited WM increases in the anterior thalamic radiations, which are generally related to executive functions, memory, behavior-planning [276,277], gait stability, and movement speed [278,279]. QMT-induced WM integrity changes have also been found in both the left and right uncinate fasciculi, which are known to play an important role in emotion regulation, learning, and language functions [280,281], possibly pointing to the role of emotional features in QMT’s efficacy to mitigate dyslexia (see following Section).

In addition to functional and structural effects, QMT has been found to elicit molecular changes [274,282,283]. Venditti and colleagues [282] reported decreased salivary proNGF after 4 weeks of QMT, which correlated with increased flexibility and creativity in both adults and children. Ben-Soussan and colleagues [274] showed increased salivary proBDNF after 12 weeks of daily training, and this result significantly correlated with increased functional connectivity. Moreover, a recent study discovered a positive correlation between proNGF and proBDNF levels in healthy adults after 12 weeks of QMT [283]. As described earlier, these neurotrophins are associated with sensorimotor training and cerebellar function.

In relation to the cerebellar involvement, QMT has also shown interesting effects on timing processes. Timing is, of course, critical to many everyday activities. It has also been proposed as one of the central deficits of dyslexia [284]. In a recent study, Ben-Soussan and colleagues [285] evaluated QMT effects on time perception and production among QMT practitioners, Aikido practitioners, and a control group by administering the time production task (i.e., to generate a response when a given interval has elapsed [286]). The authors showed that QMT practitioners demonstrated longer produced time durations than other groups of participants [285], suggesting that QMT has the capacity to extend the subjective perception of time. This has been interpreted as an increased awareness of the present moment and of the body [285], as well as decreased arousal, increased relaxation and enlarged size of perceived time units [287] due to QMT. In association with the changes in time production, QMT increased the functional synchronization in the frontal and temporal lobes, and improved neural synchronization in the cerebellum, all brain areas strongly associated with timing ability [288,289].

Since timing is one of the cognitive functions affected in dyslexia [28,32,140,148,290,291], and QMT has been proven to be capable of inducing functional, structural, and molecular changes associated with improved cognition and well-being, it should not be surprising that this whole-body MM sensorimotor training has also been studied in the context of other neurodevelopmental and neurodegenerative disorders [249,292,293]. In the next section, we will describe two studies that employed QMT among people with dyslexia and whose findings may imply the potential of QMT to serve as a remedial program for the disorder.

### 5.3. Quadrato Motor Training in Dyslexic Individuals

The first study to evaluate QMT in relation to adult dyslexia was Ben-Soussan and colleagues in 2014 [249]. Due to the important role of the cerebellum and cerebellar alpha power in voluntary action [288,294,295] and its aforementioned involvement in language and reading [101,110,140], they hypothesized QMT would increase cerebellar alpha power and, in turn, improve reading performance [249]. The authors compared dyslexic and normal readers in a magnetoencephalography (MEG) study investigating potential QMT-related modulation of cortical and cerebellar alpha power and coherence. MEG was used due to its ability to detect the source of signals stemming from the cerebellum, thus enabling a direct investigation of the cerebellar role in different reading tasks. Recruited participants were diagnosed with dyslexia by a clinical or educational psychologist and had a documented history of reading difficulties. Participants underwent three different experimental phases: pre-training, training, and post-training. In the pre-training phase, MEG data and cognitive measurements (i.e., reading test, category-based fluency task, letter-based fluency task) were collected, followed by a session of QMT. In the training phase, daily QMT sessions (7 min long) were performed by the participants themselves for 27 consecutive days. Finally, in the post-training phase, the same measurements were collected as in the first phase.

Ben-Soussan and colleagues [249] demonstrated that the pre-training cerebellar alpha activity, which was lower among the dyslexic participants (compared to controls), improved after the 4-week QMT training phase, and this activity was only enhanced among the dyslexic but not the control, participants. Moreover, unsurprisingly, the dyslexic group displayed lower pre-training scores in the letter-based fluency task compared to controls, but, more interestingly, the group discrepancy disappeared after 4 weeks of daily QMT, suggesting that QMT helped normalize performance on this task [249]. It is relevant to note that a test–retest effect was avoided by testing with different word lists pre- and post-training. In addition, no differences were identified between groups in semantic fluency, either before or after training, a pattern that supports the view that dyslexia is more related to phonological than to semantic impairment [296]. The aforementioned increase in dyslexic participants, after 4 weeks of QMT of the cerebellar alpha power, was located in the culmen [249]; this area has been previously reported to be related to language processing [101,110]. It is worth mentioning that QMT was also reported to increase limbic-frontal connectivity mediated by alpha, which is involved in the adaptive behavioral response, self-efficacy and emotional balance [230].

Ben-Soussan and colleagues [249] further reported that interhemispheric alpha coherence was higher in the dyslexic group compared to the control group, both in the pre- and post-training phases, which they suggest may be attributable to a compensation mechanism utilized by dyslexic individuals [297]. In addition, changes in the temporal alpha coherence were positively correlated with improvement in reading scores in the dyslexic group but not among the controls, indicating the relevance of this frequency band in dyslexia. In sum, the authors suggested that “*both the deficient cerebellar alpha power and possibly compensatory alpha coherence may be connected to the cerebellum’s role as a generator of alpha activity, and that sensorimotor training may lead to cerebellar plasticity, which could eventually rebalance the system*” [249] (p. 9). Accordingly, the particular combination of gross motor training and cognitive training provided by QMT may be able to increase cerebellar alpha activity among dyslexic individuals, thereby improving their reading performance; if confirmed, this would further support the notion of a relationship between cerebellar functioning and reading, especially reading speed and accuracy impairments.

The second study of QMT in adult dyslexia is particularly promising because it explored the ability of QMT to induce neuroplasticity processes in a dyslexic participant. As previously described, training-induced changes in brain connectivity and neuroplasticity (which, in turn, elicit changes in cognitive responses) are mediated by neurotrophins like proNGF and proBDNF. Such neurotrophins have previously been shown to be altered in different learning disabilities [298]. Thus, Verdone and colleagues [293], based on previous QMT research, performed a longitudinal case study examining whether ten weeks of QMT could improve reading speed and accuracy by stimulating neuroplasticity as evidenced by variations in proNGF and proBDNF levels. The participant, a 20-year-old Italian male, had an extensive history of reading and writing difficulties and was eventually diagnosed with developmental dyslexia, dysgraphia, and dysorthography. Salivary samples were collected pre-training and after four and ten weeks of training. The same was done for the cognitive tests utilized in this study: the “One Minute Reading Test” [249], consisting of a written list of increasingly difficult Italian words to be accurately read in 1 min, and the “Alternate Uses” task [229,299], a widely used assessment of divergent thinking and verbal fluency. After the salivary sample collection and cognitive testing, the QMT procedure was explained to the participant and performed the first time in the laboratory. QMT sessions for the duration of the 10 weeks were conducted at home. After the first four weeks of training, the researchers detected an increase in the number of accurately read words; this change became even more significant after the whole 10-week period. Moreover, the participant performed better in the verbal fluency component of the Alternate Uses task after four weeks of QMT, reflecting a higher total number of ideas after the training. Notwithstanding, the flexibility parameter (i.e., the tendency to generate more heterogeneous responses), did not improve after training. Finally, the authors reported differential increases in levels of proNGF and proBDNF in the salivary samples of the participant [293], with an increase in proNGF levels detectable after 4 weeks of QMT, and a proBDNF increase detected after 10 weeks of training. This finding led the authors to suggest that the reading improvement in accuracy and speed reported in this study may have been mediated by the proNGF increase at 4 weeks or by the combined proNGF and proBDNF increase at 10 weeks [293]. These results appear consistent with the previously identified QMT-induced neurotrophic effects of proNGF increase after 4 weeks of training [282] and in proBDNF after 10–12 weeks of training [274].

A final study examined the effects of time perception of a month of QMT or verbal training on time production in normal readers compared to dyslexic readers [300]. While 10 dyslexic participants performed the QMT, normal readers were randomly assigned to either QMT (*n* = 9) or Verbal Training (VT; identical cognitive training with no overt motor component and only verbal responses, *n* = 10). Results showed that, following a month of QMT, longer time production was found among the females of the dyslexic group, compared to the normal reading females. This has been suggested to be related to enhanced attention resulting from QMT [300].

Taken together, these studies show that QMT can be beneficial for improving reading skills even though this particular training practice relies on functions and abilities (e.g., response inhibition and spatial cognition) that are not necessarily directly related to reading. Such improvements in reading could be mediated by the potential of QMT to facilitate cerebellar activity and promote neuroplasticity, leading to improved timing ability that positively affects motor and cognitive symptoms of dyslexia.

## 6. Discussion

The definition of developmental dyslexia as a “deficient literacy acquisition” [9], although very useful, seems insufficient to fully encompass the various difficulties encountered by dyslexic individuals. All of those “side-impairments” of dyslexia examined before appear to be associated with different, yet related, cognitive and affective functions of the cerebellum. This similarity has led many researchers to consider these deficits not “side” at all [12,13,70]. Notwithstanding, attention to the cerebellum in remedial intervention programs has not gathered an equal momentum compared to phonological training, the remediation program that appears to offer more consistent results compared to sensorimotor training [301]. An integrated view of dyslexia comprising of bodily, cognitive and emotional aspects has yet to be reached.

Nonetheless, we have presented some encouraging findings related to a specific form of sensorimotor training, Quadrato Motor Training (QMT). QMT managed to improve reading skills and timing-related abilities among dyslexic participants, thereby highlighting the importance of cerebellar and molecular changes correlated with these improvements in the dyslexic brain. These results, consistent with the previous literature related to this training, show its ability to alter cerebellar oscillatory activity and, over an extended time frame, to induce changes in neurotrophic factors and to elicit neuroplasticity. These results provide additional support for the relevance of cerebellar involvement in dyslexia impairments.

### 6.1. Remedial Advantages of The Quadrato Motor Training

Given the evidence of the potential efficacy of QMT in remediating symptoms of dyslexia, what is the difference between QMT and other sensorimotor training? One answer to this question could lie in the specific structure of this training. Firstly, participants need to convert the verbal instructions of the QMT into correct movements within the Quadrato space. To do so, participants must have a good understanding of their spatial location within the environment, as well as the ability to retain and manage information “online” during the training (i.e., by waiting when instructed to and then move only in the right direction). In addition, participants must remain focused on the given instructions for several minutes and inhibit their natural tendency to make habitual and automatic movements. These features require sustained attention and inhibitory control [302,303], thus dividing attentional efforts between body, emotional responses and space. This activity of QMT, with its involvement of cognitive, emotional and motor functions, led to the notion that it may integrate and balance communication between cognitive, emotional, and motor areas in the brain, thus helping to monitor, modify, and ultimately strengthen cognitive control processes [17]. Given that one of the main impairments of dyslexia is related to error monitoring and control processes [69,70], QMT’s strengthening these processes (i.e., via its instruction to wait for instructions and inhibiting incorrect movements) together with its focus on motor coordination (i.e., moving in the axis to which one was instructed) could be the main reason for the identified improvements among dyslexic participants after this whole-body sensorimotor training and could be one theoretical reason for considering the QMT a threefold intervention.

Further, it is also noteworthy, in the context of an integrated view, that the beneficial effects of the QMT are not only restricted to motor functions or “cool” executive functions. Across different studies, QMT has been strongly linked to internally oriented attention, increased mindfulness, and altered states of consciousness as well as to increased creativity among healthy school-aged children [244,260], demonstrating its capabilities in improving affective processes and emotional well-being [243]. Finally, while improving reading and writing is, and should remain, the most important aim of any intervention in dyslexic participants, improving also well-being and self-efficacy may be crucial for the overall quality of life in participants affected by dyslexia.

These findings may suggest that the QMT-induced changes in cerebellar oscillatory activity and neuroplasticity discussed earlier in this work could positively impact not only dyslexic individuals, but those affected by Cerebellar Cognitive Affective Syndrome as well (for a review, see [147]).

### 6.2. The Quadrato Motor Training within the Framework of the Sphere Model of Consciousness

To better understand Quadrato Motor Training and its effect on dyslexia, and to foster a more global perception of dyslexia as a condition affecting diverse aspects of the human experience [88,304], it is important to discuss the QMT within the framework of the Sphere Model of Consciousness [305,306,307,308,309]. The SMC is configured into three main axes—time, emotion, and self-determination—that represent the features of human experience. Considered one of the most advanced current neuro-phenomenological models of consciousness, SMC highlights the relevance of self-determination as a driving force enabling change when applied to the motion/emotion/cognition triangle in the context of learning [17], as in the case of motor control, regulation of emotion and cognitive monitoring.

**Figure 4:**
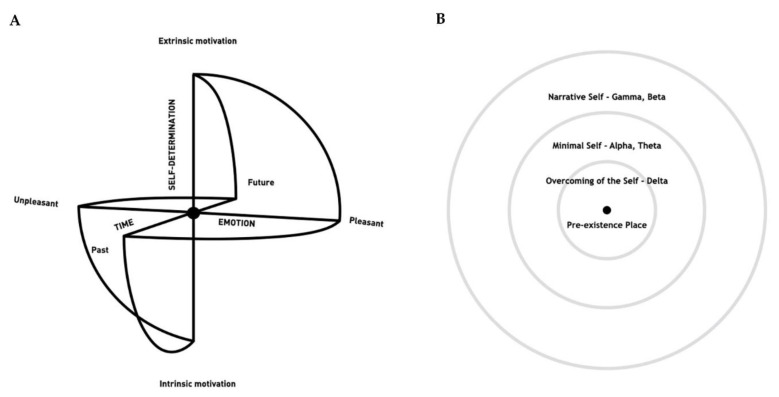
The Sphere Model of Consciousness (**A**) and its three types of Self (**B**); Narrative Self (outermost circle), Minimal Self (middle circle) and Overcoming of the Self (innermost circle) with the associated prevalent frequency bands. Adapted from [305,306,309].

In addition, the SMC posits three different types of Self which are represented as concentric circles around a centre (Figure 4). The Narrative Self relates to autobiographical memories, projections into the future, conceptual contents, and continuous awareness of personal identity; it is the outermost concentric circle of the model and the most common type of self we experience in our daily lives. The Minimal Self is illustrated as the middle concentric circle of the sphere and relates to the awareness of the body as a sensorimotor unit, the embodied selfhood anchored in the “here and now”, and non-conceptual contents.

The QMT is “a priori” linked to the SMC model, it is one of its derivations, and it holds a particular and important position relative to the SMC. First, QMT is intimately related to the Minimal Self, because its protocol is associated with an ongoing second-by-second mindful bodily awareness and waiting for upcoming instructions [230,285]. Importantly, the Minimal Self and embodied movement practices, such as the QMT, have been associated with slower frequency bands, especially alpha activity (see [309,310] for reviews), which is, in turn, closely related to creativity, relaxation and internal attention [143,144,235,251,255,256,257]. Second, the QMT constitutes an efficient way of distancing oneself from the habitual Narrative Self and, through strengthening one’s connection to the body with internally directed attention, it enables one to access the Minimal Self. In doing so, QMT allows one to advance toward the center of the SMC, the rarefied state of Overcoming of the Self [305,306,307,308,309].

The “a priori” connection between QMT and the Sphere Model of Consciousness is the hypothesis that movement in space is cognitively linked to the “inner movement” along the axes of time, emotion and self-determination within the human mind [306,307,308]. Yet, a direct parallel between QMT movements along different axes and the SMC “inner movements” towards the center of the sphere has yet to be demonstrated empirically. Nonetheless, different fields of research have indicated a strong link between physical movement in the environment and “conceptual movement” along other dimensions. For example, Flash and Hochner [311] suggested that motor actions and movements are composed of elementary building blocks called “movement primitives”, which might be equivalent to “motor schemas” or “prototypes”. They posit that combinations of these fundamental movement elements may form the syntaxis of different underlying levels of movement (i.e., dynamic, neural, and kinematic). They further suggest that the syntactic rules and laws underlying the “building” of a movement could apply also to other aspects of cognition, such as perception and language [311]. Additional evidence of a link between physical movement in space and conceptual “movement” comes from studies of the relationship between time and space processing. Such studies have consistently demonstrated that temporal and spatial processing are relatively intertwined in human cognition, proving that different cognitive and perceptual dimensions can indeed influence each other [312,313,314,315].

In fact, there is a bidirectional link between conceptual and physical movement within the framework of SMC [243,308]. A particularly fascinating phenomenon related to this topic is a specific type of “conceptual movement” called mental time travel, defined as the ability to project oneself in subjective time in order to remember the past and imagine the future [316,317,318,319]. This is made possible by the tendency of humans to represent time along a spatial continuum, in which temporal durations (short/long) and time concepts (before/after, past/future) are represented from left (short/past) to right (long/future) on a mental timeline [313,320]. In this respect, one notion about the nature of mental time travel is that it may be represented within the sensorimotor systems that regulate movement [321,322,323]. Specifically, the integration of spatial and temporal information could cause an association between mental time travel and actual movement in the environment. Indeed, just as bodily movements in space seem to influence the temporal locus of mental activity [324], so too does mental time travel have an influence in the dimension of physical movement (i.e., participants’ arm movements were attracted to different directions in the space if they were thinking about the past or the future) [325]. Mental time travel may be thus “*grounded in the embodiment of spatiotemporal information in a bidirectional manner*” [324] (p. 1). Moreover, mental time travel appears to be impaired in different conditions such as neglect [326], autism [327], old age [328], schizophrenia [329], and brain lesions [330], indicating a possible relationship between other, more specific impairments, and the act of these mental/conceptual movements. Taken together, this evidence propounds that, considering the SMC, the physical QMT movements could be tightly linked to “conceptual movements” among the types of Self and toward higher states of consciousness. Future studies should investigate this proposed relationship and explore its explanatory potential in a wider context, especially with regard to disorders and impairments associated with deficits in these types of “movements”; it may also be useful to explore movement in additional dimensions of the human experience, such as emotion [88,304], and intentionality [230,306,307,308].

In summary, QMT has demonstrated the ability to improve reading in dyslexic individuals and to elicit functional, structural, and molecular changes in association with this improvement. Being a specific form of MM, the QMT can affect not only the dyad motion-cognition but can add an additional dimension of effect to this two, reintegrating the emotional aspect in this often-dominating duality. Moreover, QMT has the practical advantage of being a relatively easy and short training (around 7 min) that can ultimately be practiced anywhere, requires limited space, and, after a few practice sessions with a specialized trainer, can be performed in everyday life without further assistance. From a motor point of view, the movements are simple and practicable, accessible from childhood to late adulthood, and relevant to both healthy and most clinical populations. In addition, it can easily be quantified in terms of accuracy and reaction time, thus providing helpful quantitative data for experimental explorations. For these reasons, QMT may serve as a powerful, relevant, and ecological tool for promoting an integrated well-being affecting the triad motion cognition and emotion (as shown in Figure 4) and possibly even remediating different functionality that has been compromised by neurodevelopmental and neurodegenerative diseases.

## 7. Conclusions, Limitations and Future Directions

Even though the findings we report are compelling, several limitations should be acknowledged. First of all, this is a scoping review, not a systematic review, so it is primarily meant to assess the extent of the available evidence, organize it, and identify gaps. Secondly, while presenting the proposed beneficial effects of QMT, we reported only three published studies that utilized only a few behavioral measures of reading and timing abilities. Moreover, the reading measures employed were limited to reading speed and accuracy and not addressing other important variables, such as reading comprehension. Nonetheless, these studies employed both functional and neuroanatomical indexes that indicate the profound effects this specific training appears to have on the brain, in general, and especially on the cerebellum of dyslexic participants, specifically. Notably, these effects are consistent with the previous literature on the behavioral, neural and molecular changes elicited by QMT. Nevertheless, the effects should be further investigated among participants with dyslexia utilizing a more detailed and comprehensive battery of tests and measures. In a similar way, future studies are recommended to explore the possibility of performing a randomized controlled trial in order to evaluate the effects of QMT on reading abilities and on other typical impairments of developmental dyslexia in a strictly controlled fashion. For a more comprehensive understanding of the effects of QMT on dyslexic individuals, we should also examine different facets of the cerebral and cerebellar functioning in concert, taking into consideration motor, emotional, and cognitive measures [17,18] and relating these examinations to existing models of dyslexia. While the studies we included in this overview surely contain some common limitations, such as small numbers of subjects and partial measures, nevertheless, their results suggest that future explorations are warranted. Future studies should examine in greater detail, with standardized methods including several more reading variables, the potential beneficial effect of QMT on a large cohort of dyslexic participants and compare it with other physical training that differ in features.

The connection between specific vectors of movements and their effect on emotion and cognition, in parallel to their psychophysiological effects, should be empirically examined. We are currently working in these directions. Finally, considering the discussed three-fold view, we must also seriously consider and study the far-reaching connections that the brain and the cerebellum have with autonomic and endocrine systems. As Dum and colleagues [331] highlighted, multisynaptic circuits link movement, cognition and emotion to the function of the adrenal medulla, mediating the effect of psychological states on the functioning of the whole body.

In conclusion, many educational programs, in general, and especially when taking into consideration learning disabilities and dyslexia, would benefit from a more integrated approach. Future studies should continue studying human development and disorders with constant attention to a holistic perspective, one in which cognitive, emotional, and motor development are fundamentally intertwined.
